# The Shifting Boundaries of Sustainability Science: Are We Doomed Yet?

**DOI:** 10.1371/journal.pbio.1001344

**Published:** 2012-06-19

**Authors:** John H. Matthews, Frederick Boltz

**Affiliations:** Center for Conservation and Government, Conservation International, Arlington, Virginia, United States of America; Imperial College London, United Kingdom

In this issue of *PLoS Biology,* Burger and colleagues make several important contributions to the discourse of sustainability science, recalling limits of human economic and population growth derived from macroecology and physical principles [1]. We agree with many of the points offered in their paper in this issue and with those in the paper by Brown and colleagues [2]. However, we also believe there is danger in a vision of sustainability that is overly deterministic and does not reflect the dynamic nature of the biosphere, its ecosystems, and economies. We are also concerned about the implications of framing sustainability in the language of physics rather than ecology.

Recent policy discussions in preparation for the Rio+20 Convention emphasize the concept of “green economies.” Perhaps most cogently described by microbiologist Lynn Margulis, the term refers to any theory of economics that views human economic activity as embedded within ecosystems. Green economics is often used with or in place of the more widely used term of “sustainability” or “sustainability science.” Both terms reflect a new, evolving, and diffuse discipline—or perhaps a goal approached through many disciplines, including ecology, economics, engineering, and sociology. Given the central role of ecosystems in current paradigms for sustainable development, the science of ecology is a seemingly natural home for sustainability science.

However, ecology may also present some operational limits to assessing or implementing sustainable strategies. Given how difficult it is to develop ecological experiments and test hypotheses, ecology has been described as having more in common with the earth sciences (such as geology) than other biological sciences (such as physiology or molecular biology), and much less with physical sciences such as chemistry and physics [3],[4]. Given the importance of observation and inference in ecology, making predictions about complex ecological interactions requires accepting their inherent uncertainty and thus a particular humility in drawing conclusions [5].

A reader of the Burger and colleagues paper [1], for instance, might assume that the logical endpoints for its arguments are either an imminent global economic collapse triggered by stringent natural resource scarcities or catastrophic human population decline in a forceful realignment with global carrying capacity. These are dire options, with no realistically actionable response, and a reader would be forced to either reject the initial assumptions or to despair, neither of which is a useful motivational force for positive change.

Moreover, while we believe that heightened concern is warranted and that these endpoints are possible, we also believe there is evidence that they can be avoided or mitigated. Predictions made on similar first principles have been put forward repeatedly in the past (e.g., [6]–[8]), and rigidly materialist approaches to social and economic change often underestimate the flexibility and resilience of human economies and societies [9]. To date, technological advances such as increases in agricultural productivity spurred by the prospect or reality of scarce primary inputs (land, water, nutrients, energy), shifts in economic valuation, and policy-based human behavioral change, such as the actions under the Montreal Protocol to reduce tropospheric concentrations of ozone-depleting gases, have avoided or delayed our transgression of perceived thresholds in the Earth system [10],[11]. While we cannot assume that there is an equivalent to Moore's Law of semiconductor capacity for natural resource management [12] or have faith that efficiency and innovation alone will save us, we can credibly assume that the existential imperative for human adjustment and adaptation will prompt us to correct our seemingly disastrous course.

As a result, we believe that sustainability itself must rest on a broader foundation, particularly if we posit that sustainability science encompasses socioeconomic development, which requires the mobilization of natural resources in new ways to sustain and improve human well-being. Here, we describe several potential gaps in sustainability science, as well as evidence for what we hope is useful optimism that emerging economic paradigms are becoming more ecologically sensitive.

## Can Economies Achieve Ecological Stability?

The term green economy references a major point of difference with sustainability science by suggesting that economies are embedded in dynamic, evolving ecosystems rather than existing in steady-state conditions. The distinction is significant; ecosystems are not unchanging or fixed but dynamic, often cyclical, and capable of evolution, transformation, and reengineering by species other than humans [13].

Ecosystems are also not isolated or fully self-contained; the laws of thermodynamics may not be heuristic for assessing sustainability at “all spatial and temporal scales” [1], particularly local scales. Thermodynamic relationships are probably most revealing as global rather than local processes given that the Earth, all ecosystems, and socioeconomic networks are thermodynamically open rather than closed. Applications of physical laws to complex biological and social systems are often challenging (e.g., [14]).

## Management and Manipulation of Ecosystems: The Consolation of History

Global economic forces and high population density characterize the current period of natural resource exploitation, but we have long influenced ecosystems in significant ways, even when we had little in the way of global trade or population pressure. For instance, a preponderance of evidence suggests that humans contributed to the extinction of many large mammals in North and South America following the Bering land bridge migration beginning about 12,000 years before present (BP), as well as of large fauna across the Pacific islands, Madagascar, and New Zealand [15]. Hydrologists have recently posited that Native American land management practices altered the dominant geomorphological features of eastern North America's mid-Atlantic rivers in the pre-Columbian era [16],[17]. Even many aspects of global trade considered new are primarily a matter of the extent and speed of change rather than novelty per se. Chinese consumption of American ginseng in the 17th and 18th centuries, for instance, almost drove the species to extinction in the Appalachian mountains [18]. Iberian forests have yet to recover from the overproduction of wool during the 16th century, while the legacy of unsustainable farming practices in ancient Greece persists as degraded topsoils today [19]. With few exceptions, current human behavior differs from the past primarily as a matter of degree—one that merits concern at global aggregate levels, but does not present novel scenarios of local overconsumption per se.

Certainly not all long-term human impacts have been negative. Intensive rice agriculture began in the Yangtze basin about 8,000 years BP, a sustainable model for agriculture by any reasonable standard [20]. The extensive water infrastructure network around Chengdu, China, has diverted part of the Min River through the Dujiangyan for both flood control and irrigation without restricting fish connectivity since 256 BC [21], while some forests in India have been actively managed by surrounding communities for even longer periods [22].

## Sustainability and Shifting Cycles: Macro-, Meso-, and Microecology

While organismal behavior (especially by humans) has profoundly altered many, if not most, ecosystems, most significant shifts in biogeochemical cycles and ecosystem qualities occur for abiotic reasons. The amount of water on earth, for instance, has declined in absolute terms about 26% since the beginning of life on Earth 3.5 billion years BP [23], but the relative balance between fresh and salt water evolves much more rapidly, normally in response to glacial-interglacial cycling. During the last glacial maximum about 20,000 years BP, glacial area extent was about 40 million km^2^, compared to about 17.5 million km^2^ today, representing many times more fresh water than now present, with sea levels over 100 m lower than currently extant [24]. Most of these transitions occurred relatively rapidly—in decades to centuries, but occasionally over sub-decadal periods—and are thus quite relevant to human lifespans [25]–[27]. Even the Holocene (∼the past 12,000 years) has seen dramatic shifts in lake levels (tens to hundreds of m) and river discharges (across several orders of magnitude) unrelated to human water management, reflecting changes in precipitation regime [28]. Fire frequency and severity for forest and savannah ecosystems are often connected to precipitation patterns [29]. These shifts have had important implications for human water management regimes, agricultural patterns, and urban densities, and pre-Columbian civilizations in the Americas excelled at developing innovative engineering approaches to manage such shifts in variability [30]. Sustainability over decadal to century timescales must be grounded in adaptive, flexible management that reflects many non-stationary aspects of human, climate, and biogeochemical conditions [31].

## Innovation, Reorganization, and Efficiency

Humans have long caused irreparable harm to ecosystems, driven species to extinction, and have in turn endured major shifts in biogeochemical cycling. We agree that such incidents are avoidable and unacceptable and that the magnitude of current trends must not be dismissed. Humans have also developed ingenious and novel ways of making resource use far more efficient or exploiting new types of resources. Obvious developments here include the invention of agriculture and the domestication of wild plant and animal species, of course, but humans have also been innovative in energy development (wood, wind, coal, petroleum, hydropower, biofuels, geothermal, biogen, nuclear, solar, and wave power), the development of synthetic chemical fertilizers in the 19th century, and the discovery of modern antibiotics in the 20th century. Other innovations have been organizational, such as the development of cities in the Levant and east and south Asia, the birth of modern experimental science, and the transition from family-tribal-moeity structures to multiple scales of governance (including corporate, national, international, and global government structures and institutions).

Some responses to economic and environmental change defy the longstanding predictions of overpopulation concerns, such as the widespread trend towards declining birthrates as living standards increase [32], though the relationship between per capita energy consumption and population growth is complex [33]. While Burger and colleagues point to increasing energy consumption over the past few centuries, they disregard important shifts in the sources of energy in progressive economies [1]; the expansion of low-carbon energy sources in China, Brazil, the European Union, and other regions in recent decades marks a critical transition, and a shift from coal-fired sources of power to hydropower or wind mark very significant transformations, with important implications for ecological footprints. For example, over 98% of Norway's electricity is derived from hydropower [34], about 20% of Brazil's transport fuels consumption is derived from renewable biofuels [35], while China has installed to date about 61 GW of windpower, or roughly three times the generation potential of the Three Gorges Dam [36]. The development of a global environmental movement is also notable in this context, as signified by both the 1992 Rio Earth Summit (attended by over 100 heads of state and 172 governments) as well as its planned 2012 successor conference, the Rio+20 Summit, in addition to important milestones achieved under the UN biodiversity and climate conventions (i.e., the United Nations Convention on Biological Diversity [UNCBD] and the United Nations Framework Convention on Climate Change [UNFCCC]).

While these and other innovations in organization, efficiency, and technology have had unintended side effects, they also resulted in major transitions in human survivorship, resource extraction efficiency, and social and cultural organization. They were also largely unanticipated or very difficult to predict for most observers prior to their invention. Taken together, humans have demonstrated great creativity in how we use technological, social, and cultural “tools” to solve resource limitations.

## Not Doomed (Yet)

Our “adjustments” to the view of sustainability science presented by Brown and colleagues [1] are not meant to obscure or downplay absolute declines in resources such as economically valuable metals and agriculturally productive land, our heedless approach to anticipated tipping points in greenhouse gas accumulation, and ecosystem transformation and species extinction. The availability of natural resources is less of a problem than absolute limits in the Earth's ability to absorb the different outputs of economic activities, while maintaining conditions necessary for human productivity, much less the survival of humans and other species. Anthropogenic climate change is perhaps the most prominent example of these new scarcities and emerging “limits to growth.” Indeed, we attribute great merit to these cautionary appeals and to the evidence of Earth system thresholds. We argue for positive responses in behavior, technological progress, and economic realignments commensurate with the challenge of fulfilling human needs while maintaining an Earth system suitable for the long-term survival of humans and other species.

The authors ask, Can the Earth support even current levels of human resource use and waste production, let alone provide for projected population growth and economic development? They answer their question with little doubt: “There is increasing evidence that modern humans have already exceeded global limits on population and socioeconomic development, because essential resources are being consumed at unsustainable rates” [1]. We agree that our present consumptive trajectory risks surpassing perceived planetary boundaries in the safe operating space for humanity (c.f. [11]). We argue that these risks merit a paradigm shift, a global transformation—and that this paradigm shift is underway. We believe that the transition from relatively static approaches to sustainability to flexible green economies embedded in dynamic, variable ecosystems will prove to be a critical intellectual shift for humans this century.

There are reasons for cautious optimism. It is no accident that the modern synthesis of payments for ecosystem services crystallized in the developing world in Costa Rica when the scarcity of ecosystem goods and services from forest conversion was recognized as a social and economic threat [37]. Revolutionary approaches to water management such as dynamic environmental flows have evolved to address both climate variability and absolute shifts in Tanzania's precipitation regime (http://www.iucn.org/about/union/secretariat/offices/esaro/what_we_do/water_and_wetlands/prbmp_esaro/). A global policy and economic transformation attributing value to standing forest has emerged with the development of “REDD+” incentives to reduce greenhouse gas emissions from deforestation, particularly in tropical forests (c.f. [38]). Many developing countries understand that Western models of development are inappropriate if not impossible to achieve. We believe that these and other positive trends are both accelerating and permeating local, national, and global economies quickly and permanently.

## Blending Conservation and Development into Green Economies

Perhaps the most significant shifts in resource management consciousness have emerged through climate change adaptation and the recognition that institutions, infrastructure, and ecosystems have been managed on the basis of climate “stationarity,” which is the assumption that the past is an effective guide to the future [30],[39].

We suggest that ecosystems and economies should be managed flexibly for at least three non-stationary processes, including demographics, economics, and climate. A fourth non-stationarity should target research and investments that lead to increased efficiency and smaller resource footprints. Taken together, these non-stationarities fit social–ecological resilience theory quite closely. Complex and shifting human interactions with ecosystems and biogeochemical cycles can be translated into decision-making processes [40].

With increasing scientific knowledge and global awareness of emerging environmental risks, scarcities, and potential tipping points in social and ecological systems, measures are being taken to correct our flawed economic models—internalizing externalities in accounting and decision making, integrating planetary boundaries in policy discussions, and committing to reverse trends in environmental and social decline. We agree with our respected colleagues that this change is not happening at the scale or pace necessary to resolve the problem [1], and exceeding tipping points is a genuine risk. Such signal failures of resource management as the collapse of the Atlantic cod fishery in the 20th century [41] or the lack of a global carbon emissions agreement at the UNFCCC CoP15 in Copenhagen in 2009 highlight our difficulty in negotiating science, institutional change, and governance. However, we also highlight that the adaptive capacity of humanity to overcome seemingly insurmountable constraints on human development within a productive and resilient biosphere has been demonstrated at more modest scales and that this capacity for transformation exists in our interconnected global community at a scale previously unimaginable.

Science-based resource management has seen dramatic growth in sophistication in recent decades, as conservation and economic development have blended together and flexible, non-stationary management approaches have become increasingly mainstream in development banks, governments and aid agencies, and corporations. These shifts represent real advances in linking ecology to practical challenges in managing resources across multiple spatial and temporal scales.

For science to maintain a useful role with policymakers and resource managers, we must find ways to communicate in ways that can be translated into policy and practical action. Our intuition is that fear has proven to be a far less helpful means of communicating the need for positive change than hope.
